# Genetic diversity of three surface protein genes in *Plasmodium malariae* from three Asian countries

**DOI:** 10.1186/s12936-018-2176-x

**Published:** 2018-01-11

**Authors:** Suttipat Srisutham, Naowarat Saralamba, Kanlaya Sriprawat, Mayfong Mayxay, Frank Smithuis, Francois Nosten, Sasithon Pukrittayakamee, Nicholas P. J. Day, Arjen M. Dondorp, Mallika Imwong

**Affiliations:** 1 Department of Molecular Tropical Medicine and Genetics, Faculty of Tropical Medicine, Mahidol University, Bangkok, Thailand; 2 Mahidol Oxford Tropical Medicine Research Unit, Faculty of Tropical Medicine, Mahidol University, Bangkok, Thailand; 3 Shoklo Malaria Research Unit, Mahidol-Oxford Tropical Medicine Research Unit, Mae Sot, Thailand; 4 Lao-Oxford-Mahosot Hospital-Wellcome Trust Research Unit (LOMWRU), Microbiology Laboratory, Mahosot Hospital, Vientiane, Lao People’s Democratic Republic; 5 Faculty of Postgraduate Studies, University of Health Sciences, Vientiane, Lao People’s Democratic Republic; 6 Centre for Tropical Medicine and Global Health, Churchill Hospital, University of Oxford, Oxford, UK; 7 Medical Action Myanmar, Yangon, Myanmar; 8 Centre for Tropical Medicine, Nuffield Department of Clinical Medicine, University of Oxford, Oxford, OX3 7LF UK; 9 Department of Clinical Tropical Medicine, Faculty of Tropical Medicine, Mahidol University, Bangkok, Thailand

**Keywords:** *Plasmodium malariae*, TRAP, AMA1, P48/45

## Abstract

**Background:**

Genetic diversity of the three important antigenic proteins, namely thrombospondin-related anonymous protein (TRAP), apical membrane antigen 1 (AMA1), and 6-cysteine protein (P48/45), all of which are found in various developmental stages of *Plasmodium* parasites is crucial for targeted vaccine development. While studies related to the genetic diversity of these proteins are available for *Plasmodium falciparum* and *Plasmodium vivax*, barely enough information exists regarding *Plasmodium malariae*. The present study aims to demonstrate the genetic variations existing among these three genes in *P. malariae* by analysing their diversity at nucleotide and protein levels.

**Methods:**

Three surface protein genes were isolated from 45 samples collected in Thailand (N = 33), Myanmar (N = 8), and Lao PDR (N = 4), using conventional polymerase chain reaction (PCR) assay. Then, the PCR products were sequenced and analysed using BioEdit, MEGA6, and DnaSP programs.

**Results:**

The average pairwise nucleotide diversities (π) of *P. malariae trap*, *ama1*, and *p48/45* were 0.00169, 0.00413, and 0.00029, respectively. The haplotype diversities (Hd) of *P. malariae trap*, *ama1*, and *p48/45* were 0.919, 0.946, and 0.130, respectively. Most of the nucleotide substitutions were non-synonymous, which indicated that the genetic variations of these genes were maintained by positive diversifying selection, thus, suggesting their role as a potential target of protective immune response. Amino acid substitutions of *P. malariae* TRAP, AMA1, and P48/45 could be categorized to 17, 20, and 2 unique amino-acid variants, respectively. For further vaccine development, carboxyl terminal of P48/45 would be a good candidate according to conserved amino acid at low genetic diversity (π = 0.2–0.3).

**Conclusions:**

High mutational diversity was observed in *P. malariae trap* and *ama1* as compared to *p48/45* in *P. malariae* samples isolated from Thailand, Myanmar, and Lao PDR. Taken together, these results suggest that P48/45 might be a good vaccine candidate against *P. malariae* infection because of its sufficiently low genetic diversity and highly conserved amino acids especially on the carboxyl end.

**Electronic supplementary material:**

The online version of this article (10.1186/s12936-018-2176-x) contains supplementary material, which is available to authorized users.

## Background

*Plasmodium malariae*-related incidents are mostly observed in endemic areas [1] and have lower prevalence than *Plasmodium falciparum* or *Plasmodium vivax* infections [2]. Several studies involving sophisticated molecular detection methods have revealed more accurate picture of *P. malariae* infection indicating much higher prevalence of the disease than previously expected in many regions of the world [3–5]. Moreover, *P. malariae* infection often displays low parasitaemia and can occur as mixed infection involving *P. falciparum* or *P. vivax* [6]. Similarly, *P. malariae* infection can remain asymptomatic for longer period of time and lead to increase in mortality by causing chronic nephrotic syndrome [7–13]. Hence, *P. malariae* is an important species that should be included in malaria elimination programmes [5]. As an important part of malaria elimination programme, extensive studies are being carried out focusing on potential molecular targets for development of effective vaccines against all forms of human malaria. One of the main surface proteins used as targets for development of vaccines is merozoite surface protein 1 (MSP1), for which the genetic diversity of its corresponding *msp1* gene has been previously analysed for *P. malariae* [14].

In this context, the investigation was focused on three important antigenic proteins found in *P. malariae* namely thrombospondin-related anonymous protein (PmTRAP), apical membrane antigen 1 (PmAMA1), and 6-cysteine protein (PmP48/45) which are known to play significant role in developmental stages of the parasite. TRAP is a sporozoite surface protein essential for sporozoite gliding, motility, and hepatocyte invasion [15–17]. This protein was utilized as a pre-erythrocytic vaccine target against *P. falciparum* (ChAd63-MVA ME-TRAP), which could reduce burden of the liver parasite by 79–84% [18]. Likely, AMA1 is a merozoite surface protein that plays a significant role in red blood cell invasion [19–24]. Using AMA1 as blood stage malaria vaccine showed 64.3% efficacy against clinical *P. falciparum* malaria [25]. P48/45 is a gametocyte antigen required for fertilization of gametocytes [26]. Moreover, P48/45 has been regarded as a promising candidate for vaccine development by transmission blockage of gametocyte in malaria vector [27].

Genetic diversity arising through antigenic polymorphisms can cause change in expression of critical epitopes resulting in reduction or even complete loss of vaccine efficacy. The success of an effective long-term vaccine development is, therefore, depended on wide-scale assessment of the degree of genetic polymorphism among parasite populations in malaria endemic areas. Genetic diversity of *trap*, *ama1*, and *p48/45* genes of *P. falciparum* and *P. vivax* have been analysed and reported by multiple independent studies [28–30]. Although genome sequence of *P. malariae* has been recently reported for its evolutionary history and phylogenetic classification [31], there barely exists a study entirely focusing on genetic diversity of TRAP, AMA1, and P48/45 genes of *P. malariae*. Thus, the present study is probably the first investigation aimed at isolation and analysis of genetic diversity of genes coding for these three surface protein in *P. malariae*. In particular, population genetic of *P. malariae* was analysed based on three genes namely, *P. malariae* thrombospondin-related anonymous protein gene (*pmtrap*), *P. malariae* apical membrane antigen 1 gene (*pmama1*), and *P. malariae* 6-cysteine protein gene (*pmp48/45*). In addition, the study also includes comparative analysis of three surface proteins across six human *Plasmodium* species. Findings from the current study might contribute to crucial information needed for further understanding and potential vaccine development against *P. malariae* infection.

## Methods

### DNA samples

DNA samples were extracted from total of 45 symptomatic *P. malariae* infected patients from Thailand (N = 33), Myanmar (N = 8), and Lao PDR (N = 4) in 2002–2013 (Table 1). These DNA samples were purified from whole blood or dried blood spot on filter paper using QIAamp DNA Mini Kit (Qiagen, Germany) and then stored at − 80 °C. These samples were subjected to confirmative identification for *Plasmodium* species using polymerase chain reaction (PCR) based on *18 small*-*subunit ribosomal* RNA [32, 33] to confirm the present of *P. malariae*.

**Table 1:** DNA samples used in the study

Countries	Study sites	Collected year	No. of samples	*Plasmodium* species^a^	Results		
					*pmtrap*	*pmama1*	*pmp48/45*
Thailand	Tak	2003–2012	22	*Pm* (n = 25) *Pm* + *Pf* (n = 3) *Pm* + *Pv* (n = 3) *Pm* + *Pf* + *Pv* (n = 2)	27	32	33
	Kanchanaburi	2003–2013	11				
Myanmar	Rakhine	2009	8	*Pm* (n = 7) *Pm* + *Pv* (n = 1)	5	7	7
Lao PDR	Savannakhet	2010	4	*Pm* (n = 4)	3	4	4
	Total		45		35	43	44

^a^*Pm*: *Plasmodium malariae*; *Pm* + *Pf*: mixed *P. malariae* and *P. falciparum*; *Pm* + *Pv*: mixed *P. malariae* and *P. vivax*; *Pm* + *Pf* + *Pv*: mixed *P. malariae*, *P. falciparum*, and *P. vivax*

### Amplification and sequencing of *Plasmodium malariae trap*, *ama1,* and *p48/45* genes

*Plasmodium malariae trap*, *ama1* and *p48/45* genes were amplified using either nested or semi-nested PCR using specifically designed primers with the optimized MgCl_2_ concentrations and annealing temperatures, which were validated previously (Additional file 1). PCR products were either cloned into pGEM T-easy vector (Promega, USA) or directly purified before DNA sequencing. The nucleotide sequences of *P. malariae trap*, *ama1* and *p48/45* genes obtained in this study have been submitted to GenBank database under the accession numbers KY905247–KY905306.

### Sequence analysis

The overall analysis procedure was divided into four major sections. Firstly, the nucleotide polymorphism of *P. malariae trap*, *ama1* and *p48/45* genes were measured. Partial DNA sequences were aligned using BioEdit and then we calculated average pairwise nucleotide diversity (π), the number of haplotypes (H), and haplotype diversity (Hd) using DnaSP v5 [34]. The nucleotide diversity was also plotted using a sliding method with a window length of 60 base pairs (bp) and a step size of 3 bp using DnaSP v5. Moreover, partial nucleotide sequences were aligned with reference sequences of *P. malariae trap*, *ama1,* and *p48/45* isolated from Uganda, accession numbers LT594633.1, LT594630.1, and LT594500.1, respectively. In addition, we also aligned the nucleotide sequences of *P. malariae trap*, *ama1,* and *p48/45* with sequences isolated from Malaysia, Indonesia, and Guinea, accession numbers ERS1110317, ERS1110321, and ERS567899, respectively [31].

Secondly, the amino acid substitutions and haplotype diversity of *P. malariae* TRAP, AMA1, and P48/45 were identified. Partial nucleotide sequences of *P. malariae trap*, *ama1*, and *p48/45* were translated to deduced amino acid sequences using BioEdit and then aligned with references amino acid sequences of *P. malariae* TRAP, AMA1, and P48/45, accession numbers SCO93694.1, SCN12851.1, and SBT79956.1, respectively. The functional domains of these three proteins based on their reference amino acid sequences were predicted using InterPro [35] (Additional file 2).

Thirdly, *P. malariae* population was characterized based on three protein genes, the codon-based test of purifying selection was analysed using MEGA6 program [36]. The mean number of synonymous mutations per synonymous site (dS) and non-synonymous substitutions per non-synonymous site (dN) within each isolate were calculated using the Nei and Gojobori method [37] with the Jukes and Cantor correction. The statistical differences between dN and dS of the three genes were tested using Z-test of selection. In addition, neutral model was utilized to determine whether polymorphism took place at higher or lower frequencies than expected. Three population genetic tests of neutrality (Tajima’s D, Fu and Li’ D* and Fu and Li’ F* tests) [38, 39] were applied to the *P. malariae trap*, *ama1* and *p48/45* gene sequences.

In addition, to provide additional information that may further contribute to current knowledge of genetic polymorphism existing among different human *Plasmodium* species, the nucleotide diversity and amino acid homology of TRAP, AMA1, and P48/45 surface proteins among six *Plasmodium* species were analysed using reference nucleotide and amino acid sequences obtained from NCBI databases (Additional file 3). Maximum likelihood trees of six human malaria parasites were constructed using MEGA6 program based on amino acid sequences of TRAP, AMA1, and P48/45 [36] to show the phylogeny among six *Plasmodium* species.

## Results

### Comparative analysis of *trap*, *ama1* and *p48/45* gene within six human *Plasmodium* species

**Fig. 1:**
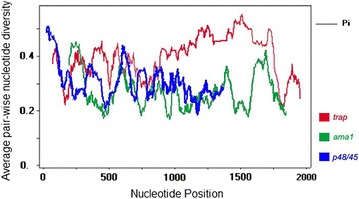
Sliding window plots of the average pair-wise nucleotide diversity (π). The nucleotide diversity of six *Plasmodium* species *trap* (red), *ama1* (green) and *p48/45* (blue) were plotted by a sliding window with a window size of 60 bp, a step size of 3 bp, and excluding sites with gaps

To provide information of diversity in *trap, ama1, and p48/45* across all human malaria species, the reference sequences for *trap, ama1,* and *p48/45* from all *Plasmodium* species were retrieved from database and analysed. The genetic variation was measured using a sliding method plot with a window length of 60 bp, a step size of 3 bp, and exclusion of sites with gaps. Results revealed overall π value for *p48/45* in range 0.19000–0.51000. However, low diversity was observed after 1 kb length with approximate π values in the range 0.2–0.3 (Fig. 1). The π diversity was also measured for *trap* and *ama1* with the overall observed range of 0.20111–0.55333 and 0.17111–0.45333, respectively. The diversity was observed with fluctuated peak along the length of both *trap* and *ama1* (Fig. 1). Multiple sequence alignment of those three corresponding surface proteins of all human *Plasmodium* species were determined. Overall sequence homology for TRAP, AMA1, and P48/45 varied from 36.44 to 89.38, 54.02–96.08, and 55.92–96.51%, respectively (Additional file 4). Among human malaria parasites, *P. malariae* TRAP, AMA1, and P48/45 high homology with *Plasmodium ovale wallikeri* TRAP (47.47%), AMA1 (71.40%) and P48/45 (63.34%), respectively. Additional comparison of *P. malariae* TRAP, AMA1, and P48/45 with *P. falciparum* and with *P. vivax* showed that the homology of *P. malariae* and *P. vivax* TRAP (45.97%), AMA1 (68.68%), and P48/45 (61.40%) were higher than the homology of *P. malariae* and *P. falciparum* TRAP (36.44%), AMA1 (56.77%), and P48/45 (58.90%), respectively (Additional file 4). Conserved amino acids were found partially along those three gene sequences. The highest level of homology across all *Plasmodium* species was observed in P48/45 with the highly conserved residues located near the carboxyl terminal. The maximum likelihood tree showed that *P. falciparum* TRAP, AMA1, and P48/45 were less related with other *Plasmodium* species. In addition, the phylogeny shows recent common ancestry of *P. malariae* and *P. ovale* based on TRAP and P48/45 (Additional file 5).

### Nucleotide polymorphism of *Plasmodium malariae trap*, *ama1,* and *p48/45*

**Fig. 2:**
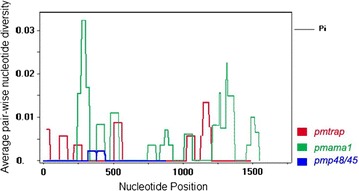
Sliding window plots of the average pair-wise nucleotide diversity (π). The nucleotide diversity of *P. malariae trap* (red), *pmama1* (green) and *pmp48/45* (blue) isolated from Thailand, Myanmar, and Lao PDR were plotted by a sliding window with a window size of 60 bp and a step size of 3 bp

Using PCR the 35, 43, and 44 isolates of *P. malariae* were successfully amplified for *trap*, *ama1* and *p48/45* genes (Table 1). The 35 isolates of *P. malariae trap* were amplified corresponding to nucleotide positions 172–1824 of *P. malariae* reference sequence accession number LT594633.1. The fragment size of *pmtrap* varied from 1653 to 1713 bp, in which the variation resulted from difference of number of repeat units. There were three patterns of 12 nucleotides repeat units, including CCAGAGGATAGA, CCAGAGAATAGA, and CCAGAGAATAGT. Among those three patterns, the most frequent repeat was CCAGAGGATAGA which accounted for 75–92% of all observed repeats. Excluding repeat regions, there were seven polymorphic nucleotide sites found among 35 samples with an average π value of 0.00169. A sliding method plot with a window length of 60 bp and a step size of 3 bp using DnaSP v5 revealed a π value in range 0.00001–0.01289 (Fig. 2). Nucleotide diversity could be categorized to 16 distinguish haplotypes with an estimated Hd of 0.919 (Table 2).

**Table 2:** Average nucleotide diversity and haplotype diversity of three surface genes of *P. malariae*, *P. falciparum*, and *P. vivax*

Gene	Haplotype diversity (n)			Average nucleotide diversity π (× 10^−3^) (n)			Average nucleotide diversity (π)
	*P. falciparum*	*P. vivax*	*P. malariae*	*P. falciparum*	*P. vivax*	*P. malariae*	*P. malariae* and *P. malariae*-liked
*trap*	0.990 (29)^a^	0.960 (63)^b^	0.919 (35)	6.120 (29)^a^	7.070 (63)^b^	1.690 (35)	0.64^d^
*ama1*	0.940 (55)^a^	0.899 (235)^b^	0.948 (43)	25.500 (55)^a^	14.880 (235)^b^	4.200 (43)	3.58^d^
*p48/45*	0.200 (10)^a^	0.484 (18)^c^	0.127 (44)	0.600 (10)^a^	1.160 (18)^c^	0.280 (44)	0.28^d^

^a^[28], ^b^ [29], ^c^ [30], ^d^ [31]

Partial nucleotide sequences of *ama1* gene were obtained from 43 *P. malariae* samples, spanning nucleotide positions 52–1632 of *P. malariae* reference sequence accession number LT594630.1. In all of the 43 *P. malariae* isolates *ama1* sequences had the same amplification size of 1581 bp. There were 17 polymorphic nucleotide sites found among 43 samples with an average π value of 0.00420. Use of sliding method plot with a window length of 60 bp and a step size of 3 bp revealed a π value in range 0.00001–0.03230 (Fig. 2). Nucleotide diversity could be categorized to 24 distinct haplotypes with an Hd value of 0.948 (Table 2).

The partial sequences of *p48/45* were successfully amplified from 44 *P. malariae* samples, covering nucleotide positions 244–1152 of *P. malariae* reference strain, PMUG01 accession number LT594500.1. Those 44 samples of *P. malariae p48/45* genes were isolated each having the same size of 909 bp. There were two polymorphic nucleotide sites having an average π value of 0.00028. Use of sliding method plot with a window length of 60 bp and a step size of 3 bp revealed a π value in range 0.00001–0.00217 (Fig. 2). Nucleotide sequence alignment analysis suggested presence of only two distinct haplotypes with an estimated Hd value of 0.127 (Table 2).

Nucleotide diversity of *P. malariae trap*, *ama1*, and *p48/45* obtained from this study were compared with the nucleotide diversity calculated for five *P. malariae* samples and two *P. malariae*-*like* (Chimpanzee infecting) parasites obtained from previous study [31]. The study result is in agreement with the previously published report of similar pattern of highest nucleotide diversity found in *P. malariae ama1* as compared to *trap* and *p48/45*. Moreover, nucleotide diversity and haplotype diversity of *P. malariae trap*, *ama1* and *p48/45* isolated during this study were compared with *P. falciparum* and *P. vivax* from previous reports [28, 30, 40] (Table 2). It was found that *P. malariae trap*, *ama1* and *p48/45* showed lower nucleotide diversity than *P. falciparum* and *P. vivax* (P < 0.05).

### Amino acid substitutions and variations of *Plasmodium malariae* TRAP, AMA1 and P48/45

**Fig. 3:**
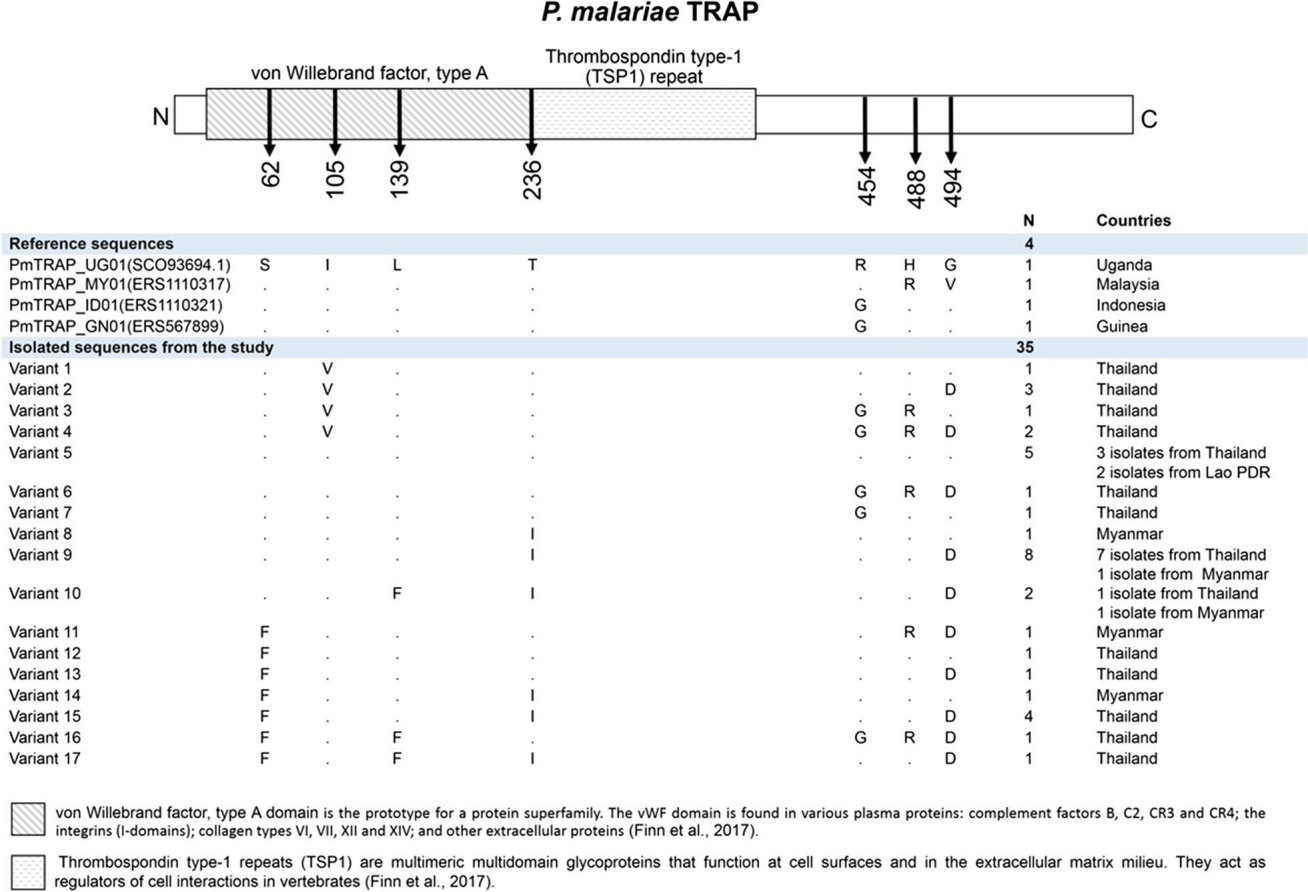
Schematic structure and amino-acid variants of *P. malariae* TRAP. *P. malariae* TRAP contains von Willebrand factor, type A and Thrombospondin type-1 (TSP1) repeat domain. The alignment of 4 reference sequences and 35 PmTRAP isolates from this study revealed 17 variant types, in which the variant 9 (SILIRHD) is the highest frequency found in this study

The isolated nucleotide sequences of *trap*, *ama1*, and *p48/45* from all *P. malariae* samples were translated to deduce their corresponding amino acids and analysed. The *trap, ama1*, and *p48/45* of *P. malariae* were intron less genes. The translated PmTRAP was found to be located on amino acid position 58–608 of *P. malariae* reference strain PMUG01 accession number SCO93694.1. The tandem repeat of 12 nucleotide unit corresponded to the three tetrapeptide unit including PEDR, PENR, and PENS, located within TSP1 domain. Number of total repeat unit among those 35 samples of *P. malariae* isolates varied from 9 to 17 repeats. The major repeat unit was PEDR which could be found from 8 to 14 units in all isolates. All of the isolates contained only one unit of PENR, except for none in one isolate from Myanmar. The repeat unit PENS also presented for 1–2 units in most *P. malariae* samples, whereas only two isolates of *P. malariae* collected in Thailand had none. Analysis of PmTRAP in non-repeat region showed seven polymorphic sites that resulted from non-synonymous mutations. Those seven polymorphic sites were 62 (S/F), 105 (I/V), 139 (F/L), 236 (I, T), 454 (G, R), 488 (H, R), and 494 (D, G). Alignment of the 35 PmTRAP isolates revealed 17 variant types, of which the variant 9 (SILIRHD) was seen in 8 out of 35 isolates, thus, making it the most frequently observed variant type among the samples (Figs. 3, 6a, Additional file 6).

**Fig. 4:**
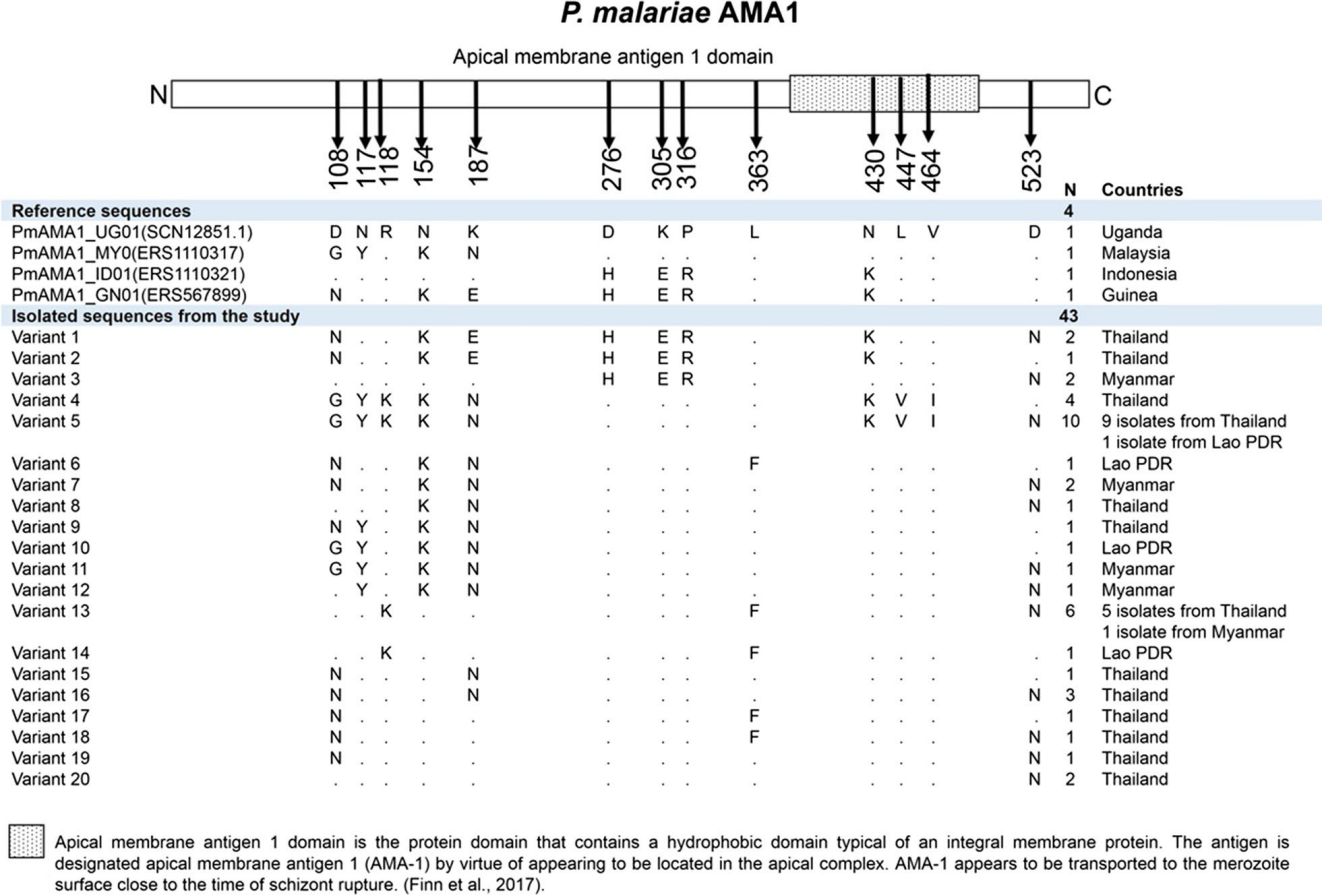
Schematic structure and amino-acid variants of *P. malariae* AMA1. *P. malariae* AMA1 contains apical membrane antigen 1 domain. The alignment of 4 reference sequences and 43 PmAMA1 isolates from this study revealed 20 variant types, in which the variant 5 (GYKKNDKPLKVIN) is the highest frequency found in this study

The translated PmAMA1 was corresponding to amino acid positions 18–544 of *P. malariae* reference strain, PMUG01, accession number SCN12851.1. There were 527 residues of PmAMA1 from 43 isolates of *P. malariae* for analysis. Sequence alignment of—PmAMA1 from those 43 isolates revealed 18 polymorphic sites at the nucleotide level, which in turn consisted of 13 non-synonymous sites and one synonymous position. The pattern of PmAMA1 polymorphisms observed in 43 *P. malariae* isolates could be classified into 20 variant types. Variant type 5 was found with the highest frequency in 10 out of 43 isolates (Figs. 4, 6b, Additional file 6). Most of the samples expressing variant type 5 were from Thailand (N = 9).

**Fig. 5:**
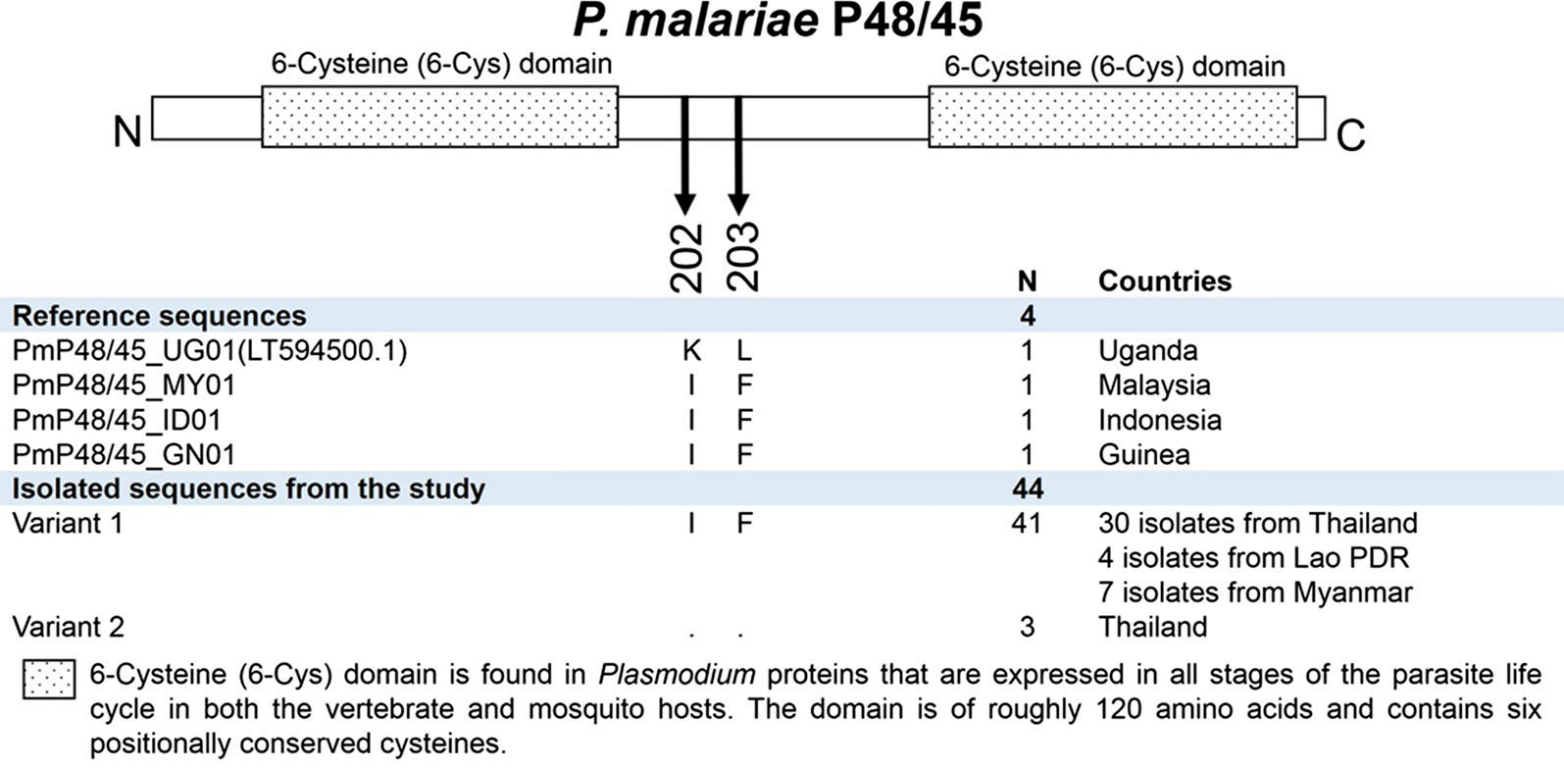
Schematic structure and amino-acid haplotypes of *P. malariae* P48/45. *P. malariae* P48/45 contains two regions of 6-cysteine domain. The alignment of 4 reference sequences and 44 PmP48/45 isolates from this study revealed 2 variant types, in which the variant 1 (IF) is the highest frequency found in this study

**Fig. 6:**
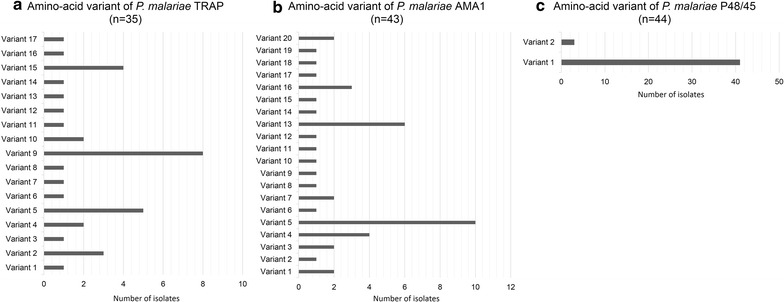
Bar chart of amino-acid variants frequency of *P. malariae* TRAP (**a**), AMA1 (**b**), and P48/45 (**c**) isolated from Thailand, Myanmar, and Lao PDR. Among these 3 proteins, P48/45 was the lowest amino-acid variation, which had 2 variants and 93.18% of 44 isolates were variant 1

The P48/45 were translated from 44 *P. malariae* samples, spanning amino acid residue 82–384 of *P. malariae* reference strain, PMUG01, accession number SBT79956.1. Total of 303 residues from 44 samples of PmP48/45 were analysed and results showed two non-synonymous sites at 202 (I, K) and 203 (F, L) (Figs. 5, 6c, Additional file 6). There were only two variant types found among those 44 *P. malariae* isolates. There were 41 samples found in variant 1 while only 3 samples of *P. malariae* exhibited variant type 2 (Fig. 5). Previous report of PmP48/45 sequences analysis from Malaysia, Indonesia and Guinea [31] contained the same haplotype pattern as variant type 1, while the isolate from Uganda [31] had the same haplotype as variant type 2.

### Genetic population testing of *P. malariae trap*, *ama1*, and *p48/45*

Population genetics of *P. malariae* was analysed based on *trap*, *ama1*, and *p48/45* gene polymorphism. The codon-based test of purifying selection was analysed using MEGA6 program. The average dS and dN values within each isolate were calculated using Nei and Gojobori method [37] with Jukes and Cantor correction. Statistical differences between dN and dS were tested with the Z–test of selection using the MEGA6 program. Results indicated that there was positive selection or diversifying selection in *P. malariae trap*, *ama1*, and *p48/45* population (Prob = 1.000, dS–dN = − 2.509 (*trap*), − 1.243 (*ama1*), and − 1.479 (*p48/45*). Besides, these three population genetic tests, the additional test for neutrality (Tajima’s D, Fu and Li’ D* and Fu and Li’ F* tests) [38, 39] was applied to the *pmtrap*, *pmama1* and *pmp48/45* sequences to determine whether polymorphism takes place at higher or lower frequencies than expected under a neutral model. Tajima’s D, Fu and Li D*, and Fu and Li F* tests using DnaSP v5 and MEGA6 were performed for *pmtrap*, *pmama1* and *pmp48/45*. The results from Tajima’s D value was 1.83835 for *pmama1* (P < 0.10), which was similar to Fu and Li’ F* test value 1.71236 (P < 0.05) (Table 3). Significantly positive values for these statistics reflect an excess of intermediate-frequency alleles, which can result from population bottlenecks, structures or balancing selections. The Tajima’s D, Fu and Li’ D* and Fu and Li’ F* tests of *pmtrap* and *pmp48/45* were not statistically significant (P > 0.10). Moreover, Fu’s Fs statistic was negative for *pmtrap* and *pmama1* which indicated population expansion of *trap* and *ama1* of *P. malariae* in the study samples.

**Table 3:** Neutrality test of *pmtrap*, *pmama1*, and *pmp48/45*

	*pmtrap* (n = 35)	*pmama1* (n = 43)	*pmp48/45* (n = 44)
Tajima’s D	1.45070	1.83702*	− 0.80011
Fu and Li’s D*	1.26400	1.24940	0.76072
Fu and Li’s F*	1.54462*	1.71102**	0.35307
Fu’s Fs statistic	− 8.379	− 8.091	0.669

* 0.05 < P < 0.10, ** P < 0.05

## Discussion

The development of modern high-throughput ultrasensitive diagnostic assays have demonstrated that *P. malariae* is more widely circulated in malaria endemic regions than previously acknowledged. Hence, this makes study of *P. malariae* essential to be included in malaria elimination programme [3–5]. Effective vaccine protecting all human malarias should be developed as part of malaria elimination programme. The lack of adequate information regarding genetic polymorphism and its underlying mechanism of the genes coding for critical surface proteins such as TRAP, AMA1, and P48/45 in *P. malariae* has limited limits current understanding of the parasite.

Genetic analysis for nucleotide and haplotype diversity suggests that *pmp48/45* has the lowest diversity among all of the studied genes. There were only two unique amino-acid variants found in PmP48/45 compare to 17 and 20 amino-acid variants observed in PmTRAP and PmAMA1, respectively. Moreover, results from comparative analysis of six human *Plasmodium* species showed lowest diversity of *p48/45* among the parasites. In accordance to these finding, highly conserved expression of *p48/45* have been previously reported in *P. falciparum* [28] and *P. vivax* [30] isolates which might be related to the critical function of this protein for transmission and oocyst development of the parasite [26]. Low variation observed in *p48/45* nucleotide sequence coding for P48/45 protein and its relatively conserved amino acid residues may be linked to the gametocyte stage where it its mainly expressed. Interestingly, this protein has been previously investigated in other *Plasmodium* species for development of potential malaria vaccine targeting transmission stage of the parasite [27].

Noticeable levels of nucleotide polymorphism observed within *P. malariae ama1* suggests that *P. malariae* may have substantial potency to exhibit genetic variation or acquire mutation on *pmama1* protein expression. In particular, most of the polymorphism were found at N-terminal position indicating possibility of directional mutation of *pmama1* gene favoring N-terminal-linked functions of the protein. The genetic diversity observed in *P. malariae trap* was comparatively lower than *pmama1*. These results are in agreement with the previously published report of similar pattern of high nucleotide diversity found in *P. falciparum ama1* as compared to *P. falciparum trap* in regions of America, Asia Pacific, and Africa [28]. This noticeable level of genetic polymorphism observed in *ama1* of six *Plasmodium* species might be related to the stage of parasite infection and functional aspect of the protein. The *Plasmodium* expresses AMA1 during merozoite stage of the parasite where it has to actively evade host immunity. Thus, acquiring genetic diversity in such crucial functional proteins can assist avoidance of host detection and subsequent neutralization of the parasite. Since AMA1 and TRAP both are expressed during active infection stages of *Plasmodium*, the evolutionary requirement level of genetic polymorphism might have been considerably higher for these genes as compared to P48/45 which is mainly expressed in gametocytes. In addition, within the *P. malariae trap*, the twelve nucleotides repeat was frequently observed for 9–17 times in the samples indicating possibility of this repeat to be used as genotyping marker.

The *pmtrap* and *pmama1* genes analysed during this study exhibited relatively more nucleotide diversity than *pmp48/45*. Both of these genes and their expression proteins have been previously targeted for development of pre-erythrocytic and blood stage vaccines against *P. falciparum* based on their immunogenic and conserved regions [18, 25]. These findings indicated presence of conserved regions in *P. malariae* TRAP and AMA1, which might be useful as potential vaccine target against the parasite. These regions containing largest amount of conserved amino acid sequences for PmTRAP (N = 35) and PmAMA1 (N = 43) were found to be located in positions 237–453 and 18–107, respectively. In addition, the nucleotide diversities observed in *pmtrap* and *pmama1* during our observation were significantly lower compared to the levels previously reported in *P. falciparum* and *P. vivax* [28, 40]. Thus, these results collectively suggests the possibility to develop malaria vaccine against *P. malariae* targeting conserved regions of PmTRAP and PmAMA1.

The ability of parasite to evolve and survive is greatly influenced by its ability to acquire genetic mutations to withstand host immunity and environmental stress [28, 40]. The results indicated higher occurrence of non-synonymous mutation in *P. malariae trap*, *ama1*, and *p48/45* genes suggesting positive or diversifying selection. In addition, the results from Z-test indicates that the polymorphisms in these genes were most probably achieved through positive diversifying selection. Such mechanisms have been reported to be favourable for the parasite to evade targeted host immune responses [28, 30, 40]. However, future study involving large number of samples from diverse geographical locations can help create clearer picture of the genetic polymorphism in *P. malariae* and their possible implications in malaria research.

In addition, the *P. malariae trap*, *ama1* and *p48/45* showed lower nucleotide diversity compared to *P. falciparum* and *P. vivax*, which might have been linked to the lower transmission rate of *P. malariae* in these geographic regions [41] and the limited number of *P. malariae* samples analysed. Nonetheless, these findings were similar to previously published data that reported low level of sequence diversity of *msp1* gene in *P. malariae* parasite as opposed to other *Plasmodium* species [14].

## Conclusions

In summary, present investigation is the first reported study revealing genetic diversity of three surface protein coding genes namely *trap*, *ama1*, and *p48/45* in *P. malariae*. Results of this study inevitability contributes to the malaria research and may potentially be a valuable resource for further vaccine development or genotyping marker.

## Additional files

**Additional file 1.** Specific primers and PCR conditions for isolation of 3 surface protein genes.

**Additional file 2.** Domain prediction of *P. malariae* TRAP, AMA1, and P48/45 using InterPro.

**Additional file 3.** Accession numbers of amino acid sequences and gene IDs of six *Plasmodium* TRAP, AMA1, and P48/45 obtained from database.

**Additional file 4.** Amino acid identity of non-cytoplasmic region TRAP, AMA1, and P48/45 within six *Plasmodium* species.

**Additional file 5.** Maximum likelihood trees of six human malaria parasites based on amino acid sequences of TRAP, AMA1, and P48/45.

**Additional file 6.** PmTRAP1, PmAMA1, and PmP48/45 variant frequencies in Thailand, Myanmar, and Lao PDR.

## Abbreviations

TRAP: thrombospondin-related anonymous protein
AMA1: apical membrane antigen 1 protein
P48/45: 6-cysteine protein
MSP1: merozoite surface protein 1
Hd: haplotype diversities
π: average pairwise nucleotide diversities
