# Supplementary material for: Genetic diversity of three surface protein genes in Plasmodium malariae from three Asian countries
Source: Malar J. 2018 Jan 11;17:24. doi: 10.1186/s12936-018-2176-x (PMC5765603; doi:10.1186/s12936-018-2176-x)
Supplement: Supplementary file 1 — Additional file 1. Specific primers and PCR conditions for isolation of 3 surface protein genes. [file 12936_2018_2176_MOESM1_ESM.pdf]

Table S1: Specific primers and PCR conditions for isolation of 3 surface protein genes.

Nested PCR condition		Name of Forward-Reverse primer	Primer sequence (5’ to 3’)	PCR condition			
				Annealing Temperature (°C)	$MgCl_2$ (mM)	No. of PCR cycle	Estimated PCR Product size (bp)
P. malariae trap	Nested 1	PmtrapF170	5’ GGAAGTATAGGAGAAGAAAACT 3’	58	3	30	1,700-1,800
		PmtrapR1	5’ TCTTCTGGTAGTTTGAATTGGT 3’				
	Nested 2	PmtrapF170	5’ GGAAGTATAGGAGAAGAAAACT 3’	60	3	35	600-700
		PmtrapR900	5’ATCATCGTTTCCACCTGGTC 3’				
	Nested 2	PmtrapF750	5’ CTTGTGGAAAGGGAACCAAA 3’	60	3	35	900-1,000
		PmtrapR1	5’ TCTTCTGGTAGTTTGAATTGGT 3’				
P. malariae ama1	Nested 1	Pmama1F22	5’ TTACTTTTGAGCACTCAATATT 3’	56	3	30	1,700-1,800
		Pmama1R1	5’ TCCATCAAAACGGGGGTAG 3’				
	Nested 2	Pmama1F22	5’ TTACTTTTGAGCACTCAATATT 3’	58	3	35	900-1,000
		Pmama1R1000	5’ TGCTCTTATTAAAATTAGCATTA 3’				
	Nested 2	Pmama1F800	5’TGTGAAGAAATTCCATCTGTTA 3’	58	3	35	900-1,000
		Pmama1R1	5’ TCCATCAAAACGGGGGTAG 3’				
P. malariae p48/45	Nested 1	Pmp4845F160	5’TGTACATAATTTAGAACCGGATA 3’	58	3	30	1,000-1,100
		Pmp4845R1200	5’ TATTATCACCTTTACTATCATCA 3’				
	Nested 2	Pmama1F170	5’ CCGGATATCCTAGAAAATAGAT 3’	60	3	35	1,000-1,100
		Pmama1R1200	5’ TATTATCACCTTTACTATCATCA 3’				
