# Supplementary material for: Genetic diversity of three surface protein genes in Plasmodium malariae from three Asian countries
Source: Malar J. 2018 Jan 11;17:24. doi: 10.1186/s12936-018-2176-x (PMC5765603; doi:10.1186/s12936-018-2176-x)
Supplement: Supplementary file 2 — Additional file 2. Domain prediction of P. malariae TRAP, AMA1, and P48/45 using InterPro. [file 12936_2018_2176_MOESM2_ESM.pdf]

Table S2: Domain prediction of *P. malariae* TRAP, AMA1, and P48/45 using InterPro. (http://www.ebi.ac.uk/interpro/) (Finn et al., 2017)

Proteins	Accession number	Predicted domains	Amino-acid positions
P. malariae TRAP	SCO93694.1	von Willebrand factor, type A	21-236
		Thrombospondin type-1 (TSP1) repeat	236-376
P. malariae AMA1	SCN12851.1	Apical membrane antigen 1 domain	380-479
P. malariae P48/45	SBT79956.1	6-Cysteine (6-Cys) domain	43-182, 296-436

FINN, R. D., ATTWOOD, T. K., BABBITT, P. C., BATEMAN, A., BORK, P., BRIDGE, A. J., CHANG, H. Y., DOSZTANYI, Z., EL-GEBALI, S., FRASER, M., GOUGH, J., HAFT, D., HOLLIDAY, G. L., HUANG, H., HUANG, X., LETUNIC, I., LOPEZ, R., LU, S., MARCHLER-BAUER, A., MI, H., MISTRY, J., NATALE, D. A., NECCI, M., NUKA, G., ORENGO, C. A., PARK, Y., PESSEAT, S., PIOVESAN, D., POTTER, S. C., RAWLINGS, N. D., REDASCHI, N., RICHARDSON, L., RIVOIRE, C., SANGRADOR-VEGAS, A., SIGRIST, C., SILLITOE, I., SMITHERS, B., SQUIZZATO, S., SUTTON, G., THANKI, N., THOMAS, P. D., TOSATTO, S. C., WU, C. H., XENARIOS, I., YEH, L. S., YOUNG, S. Y. & MITCHELL, A. L. 2017. InterPro in 2017-beyond protein family and domain annotations. *Nucleic Acids Res,* 45**,** D190-D199.
