# Supplementary material for: Genetic diversity of three surface protein genes in Plasmodium malariae from three Asian countries
Source: Malar J. 2018 Jan 11;17:24. doi: 10.1186/s12936-018-2176-x (PMC5765603; doi:10.1186/s12936-018-2176-x)
Supplement: Supplementary file 3 — Additional file 3. Accession numbers of amino acid sequences and gene IDs of six Plasmodium TRAP, AMA1, and P48/45 obtained from database. [file 12936_2018_2176_MOESM3_ESM.pdf]

Table S3: Accession numbers of amino acid sequences and gene IDs of six *Plasmodium* TRAP, AMA1, and P48/45 obtained from database.

No.	Amino acid sequences	NCBI Accession numbers	Gene IDs
1.	*P. malariae* TRAP	SCO93694.1	n/a
2.	*P. falciparum* TRAP	XP_001350088.1	814170
3.	*P. vivax* TRAP	XP_001614147.1	5473432
4.	*P. knowlesi* TRAP	XP_002259987.1	7322479
5.	*P. ovale wallikeri* TRAP	SBT40671.1	n/a
6.	*P. ovale curtisi* TRAP	SBS96280.1	n/a
7.	*P. malariae* AMA1	SCN12851.1	n/a
8.	*P. falciparum* AMA1	XP_001348015.1	810891
9.	*P. vivax* AMA1	XP_001615447.1	5474743
10.	*P. knowlesi* AMA1	XP_002259339.1	7320803
11.	*P. ovale wallikeri* AMA1	SBT35580.1	n/a
12.	*P. ovale curtisi* AMA1	SBS91605.1	n/a
13.	*P. malariae* P48/45	SBT79956.1	n/a
14.	*P. falciparum* P48/45	XP_001350181.1	814212
15.	*P. vivax* P48/45	AFB76627.1	n/a
16.	*P. knowlesi* P48/45	XP_002259885.1	7322219
17.	*P. ovale wallikeri* P48/45	SBT40052.1	n/a
18.	*P. ovale curtisi* P48/45	SBS85830.1	n/a
