# Supplementary material for: Genetic diversity of three surface protein genes in Plasmodium malariae from three Asian countries
Source: Malar J. 2018 Jan 11;17:24. doi: 10.1186/s12936-018-2176-x (PMC5765603; doi:10.1186/s12936-018-2176-x)
Supplement: Supplementary file 4 — Additional file 4. Amino acid identity of non-cytoplasmic region TRAP, AMA1, and P48/45 within six Plasmodium species. [file 12936_2018_2176_MOESM4_ESM.pdf]

Table S4: Amino acid identity of non-cytoplasmic region TRAP, AMA1, and P48/45 within six *Plasmodium* species.

	***Plasmodium* species (Accession number)**	***P. malariae***	***P. falciparum***	***P. vivax***	***P. knowlesi***	***P. ovale wallikeri***	***P.ovale curtisi***
**TRAP**	*P. malariae* (SCO93694.1)	100					
	P. falciparum (XP_001350088.1)	36.44	100				
	P. vivax (XP_001614147.1)	45.97	41.95	100			
	P. knowlesi (XP_002259987.1)	44.61	40.74	67.52	100		
	P. ovale wallikeri (SBT40671.1)	47.47	38.62	48.80	49.15	100	
	P. ovale curtisi (SBS96280.1)	45.88	39.78	48.37	47.66	89.38	100
**AMA1**	*P. malariae* (SCN12851.1)	100					
	P. falciparum (XP_001348015.1)	56.77	100				
	P. vivax (XP_001615447.1)	68.68	54.75	100			
	P. knowlesi (XP_002259339.1)	69.26	56.16	84.88	100		
	P. ovale wallikeri (SBT35580.1)	71.40	54.02	72.67	74.89	100	
	P. ovale curtisi (SBS91605.1)	70.97	54.32	72.26	74.03	96.08	100
**P48/45**	*P. malariae* (SBT79956.1)	100					
	P. falciparum (XP_001350181.1)	58.90	100				
	P. vivax (AFB76627.1)	61.40	57.43	100			
	P. knowlesi (XP_002259885.1)	60.65	55.92	83.62	100		
	P. ovale wallikeri (SBT40052.1)	63.34	59.15	63.07	61.81	100	
	P. ovale curtisi (SBS85830.1)	63.18	58.90	62.59	62.09	96.51	100
