# Supplementary material for: Genetic diversity of three surface protein genes in Plasmodium malariae from three Asian countries
Source: Malar J. 2018 Jan 11;17:24. doi: 10.1186/s12936-018-2176-x (PMC5765603; doi:10.1186/s12936-018-2176-x)
Supplement: Supplementary file 5 — Additional file 5. Maximum likelihood trees of six human malaria parasites based on amino acid sequences of TRAP, AMA1, and P48/45. [file 12936_2018_2176_MOESM5_ESM.pdf]

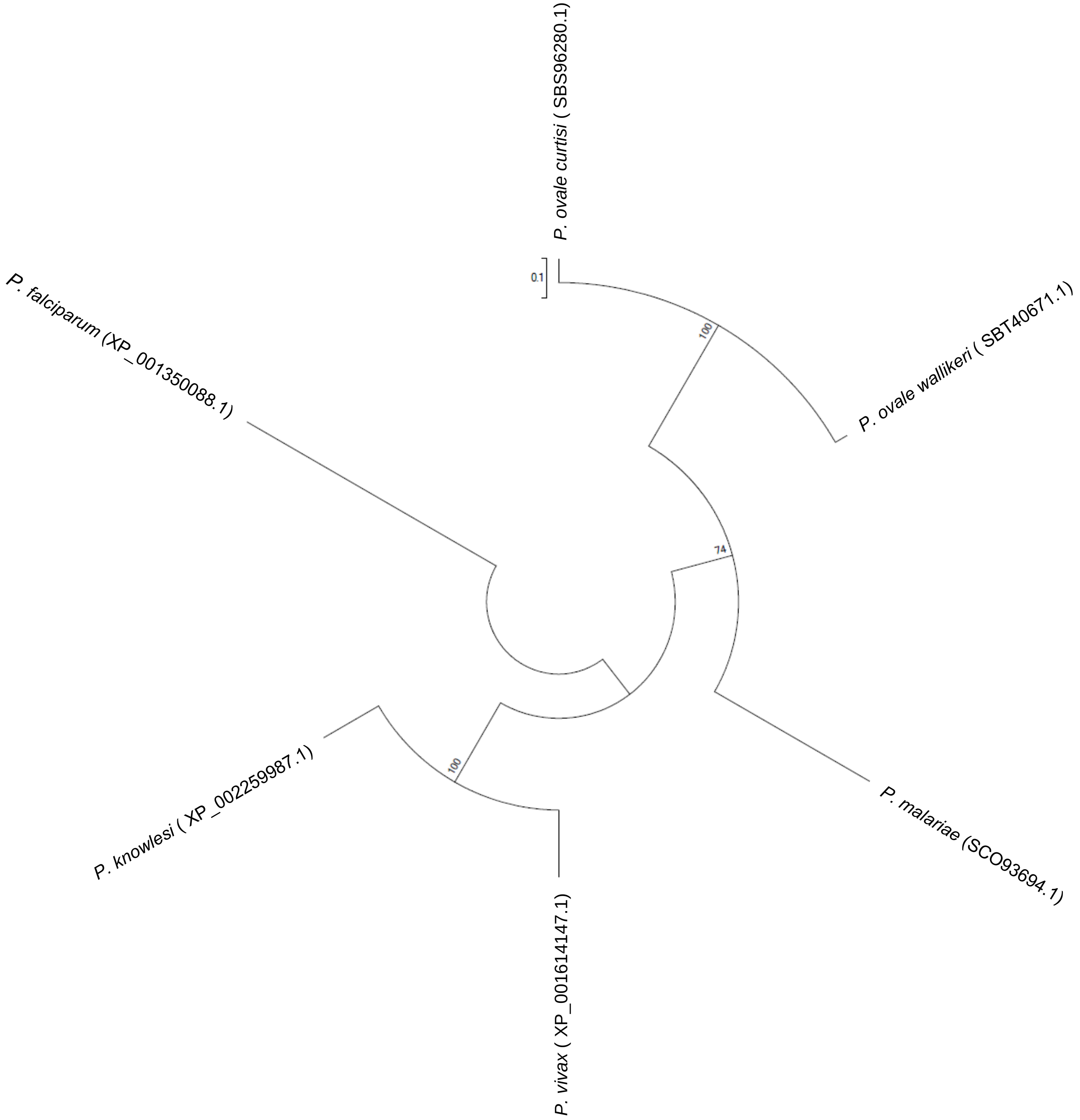

Figure S1: Phylogenetic relationship based on the TRAP sequences of six *Plasmodium* species.

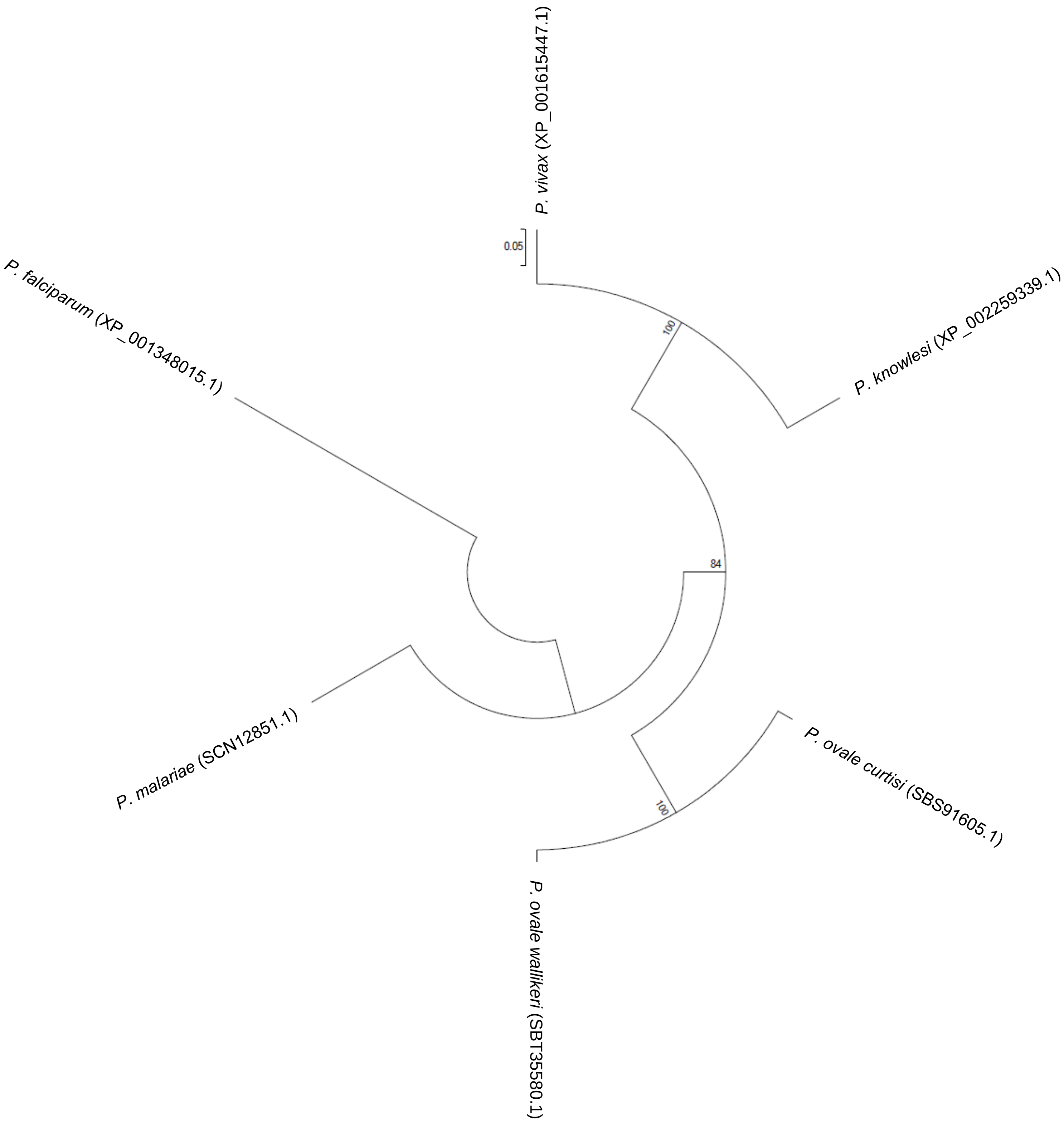

Figure S2: Phylogenetic relationship based on the AMA1 sequences of six *Plasmodium* species.

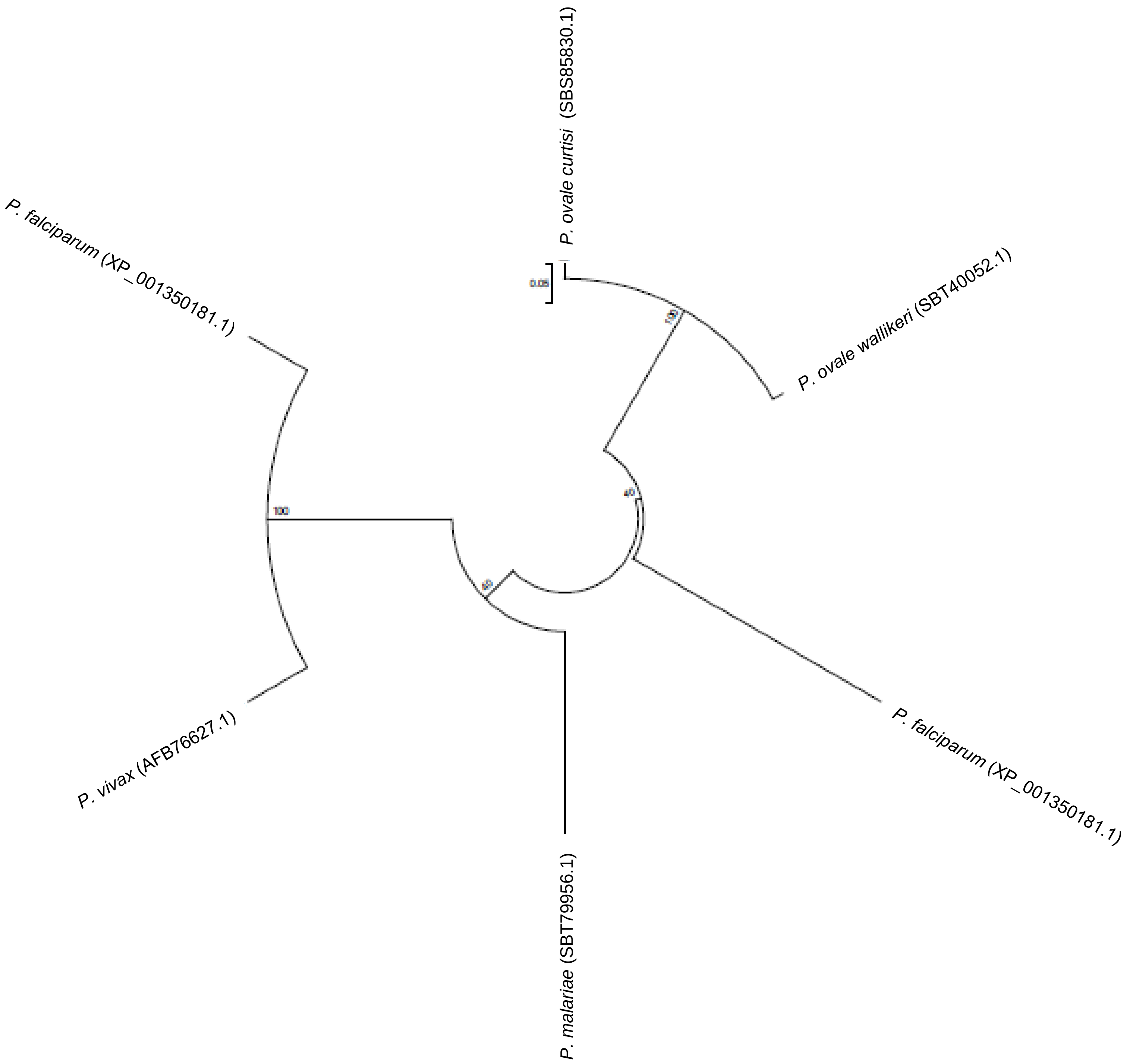

Figure S3: Phylogenetic relationship based on the P48/45 sequences of six *Plasmodium* species.
