# Supplementary material for: Genetic diversity of three surface protein genes in Plasmodium malariae from three Asian countries
Source: Malar J. 2018 Jan 11;17:24. doi: 10.1186/s12936-018-2176-x (PMC5765603; doi:10.1186/s12936-018-2176-x)
Supplement: Supplementary file 6 — Additional file 6. PmTRAP1, PmAMA1, and PmP48/45 variant frequencies in Thailand, Myanmar, and Lao PDR. [file 12936_2018_2176_MOESM6_ESM.pdf]

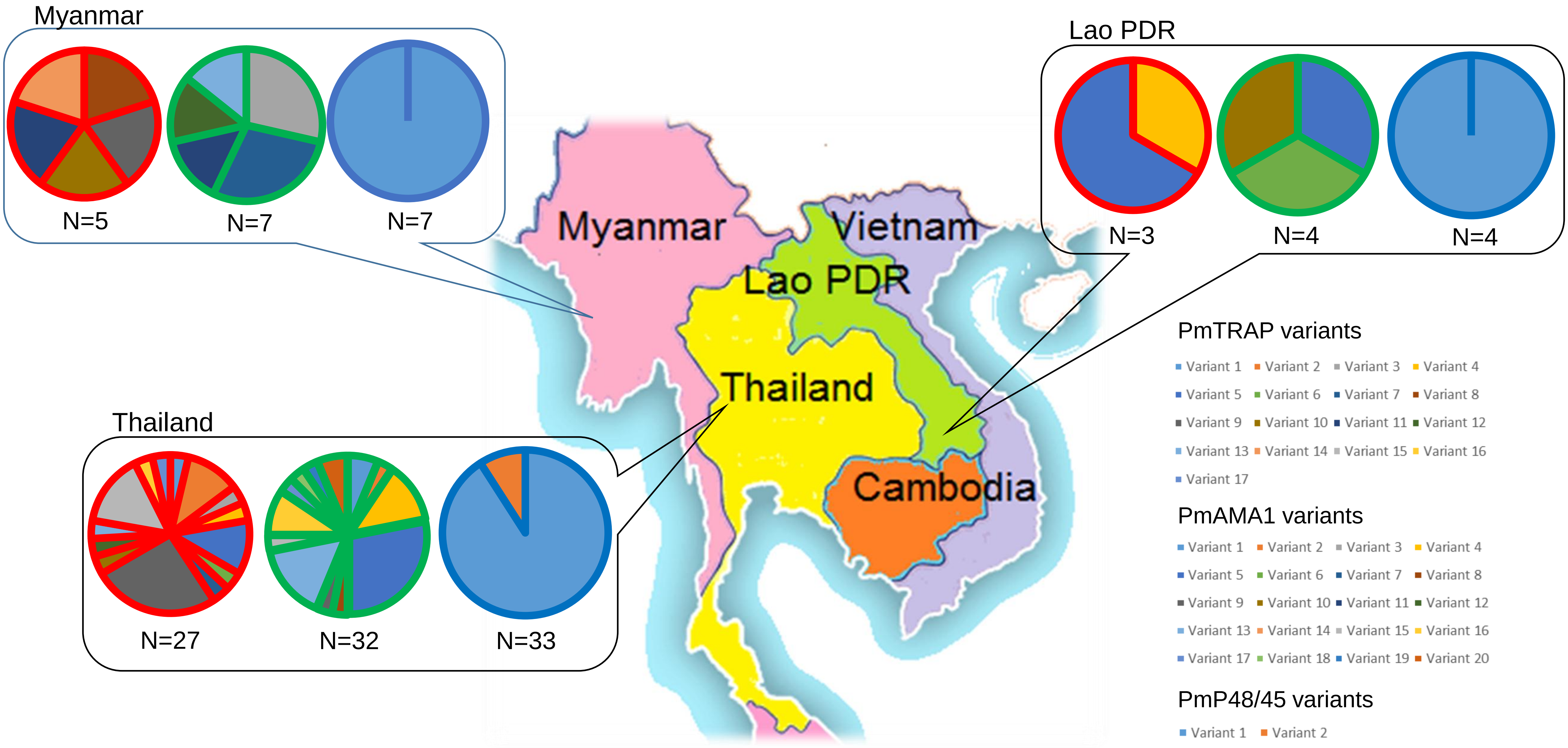

Figure S4: PmTRAP1, PmAMA1, and PmP48/45 variant frequencies in Thailand, Myanmar, and Lao PDR. Pie charts in red, green, blue circles represent the PmTRAP1, PmAMA1, and PmP48/45 variant frequencies respectively.
